# Dan Hong Injection Protects Against Cardiomyocytes Apoptosis by Maintaining Mitochondrial Integrity Through Keap1/Nuclear Factor Erythroid 2-Related Factor 2/JNK Pathway

**DOI:** 10.3389/fphar.2020.591197

**Published:** 2020-10-30

**Authors:** Ling Zhang, Yu Wang, Chang Li, Chongyu Shao, Huifen Zhou, Jiehong Yang, Yu He, Haitong Wan

**Affiliations:** ^1^ College of Life Science, Zhejiang Chinese Medical University, Hangzhou, China; ^2^ College of Basic Medical Sciences, Zhejiang Chinese Medical University, Hangzhou, China; ^3^ College of Pharmaceutical Science, Zhejiang Chinese Medical University, Hangzhou, China

**Keywords:** danhong injection, myocardial ischemia/reperfusion injury, mitochondrial function, mitochondrial morphology, apoptosis

## Abstract

Danhong injection (DHI) is used widely against cardiovascular disease in China. Recent studies have demonstrated its mitochondria-protection effect as being pivotal in treatment of myocardial ischemia/reperfusion (I/R) injury, but the underlying mechanism of action is incompletely understood. We aimed to identify the effect and mechanism of action of DHI on mitochondrial integrity and cardiomyocyte apoptosis after I/R. An I/R rat model was induced to detect the effect of DHI on myocardial repair by infarct size, apoptosis and oxidative stress. *In vitro*, H9C2 cells or H9C2 cells with nuclear factor erythroid 2-related factor 2 (Nrf2) knockdown were injured under hypoxia-reoxygenation (H/R). The effects of DHI on apoptosis, antioxidant capacity and mitochondrial integrity were evaluated by mitochondrial morphology, apoptosis rate, reactive oxygen species (ROS) generation, ATP levels, mitochondrial membrane potential, and oxygen consumption in H9C2 cells treated with H/R. The underlying mechanism of action of DHI in maintenance of mitochondrial integrity and anti-apoptosis was detected in H9C2 cells with or without Nrf2 knockdown. DHI treatment significantly decreased the infarct size, inhibited apoptosis and suppressed oxidative stress in the hearts of I/R rats. Also, DHI promoted cell survival by: an anti-apoptosis action; inhibiting ROS generation; maintaining mitochondrial morphology with increased mitochondrial length; alleviating mitochondrial dysfunction with a decreased mitochondrial membrane potential; increasing ATP levels and the oxygen-consumption rate. Moreover, the Keap1/Nrf2/JNK pathway was found to be involved in DHI reducing oxidative stress and maintaining mitochondrial integrity. We revealed a novel mechanism by which DHI protected H9C2 cells against H/R injury *via* the Keap1/Nrf2/JNK pathway and provided a mitochondrial protectant for the treatment of myocardial I/R injury.

## Introduction

Persistent myocardial ischemia causes permanent damage to the myocardium, which is destroyed and replaced by fibrous scar tissue and subsequently develops into heart failure. Timely restoration of blood flow to the ischemic myocardium limits infarct size, improves cardiac function, reduces mortality, and has become first-line treatment of myocardial ischemia. However, a considerable volume of experimental and clinical evidence suggests that reperfusion after coronary thrombosis [e.g., thrombolysis and percutaneous coronary intervention (PCI)] induces additional damage to the myocardium, and is known as “ischemia/reperfusion (IR) injury” (Ibáñez et al., 2015). Cardiomyocyte death is the main manifestation of myocardial IR injury, and is considered to be the main potential cause of ventricular remodeling and cardiac dysfunction (Rabinovich-Nikitin et al., 2019). The mechanisms underlying IR are multifactorial (Hausenloy et al., 2013). However, in general, it is accepted that mitochondrial dysfunction plays a key part in IR pathology and is the major cause of the injury and death of cardiomyocytes (Pell et al., 2016). Mitochondria occupy 25–30% of cardiomyocyte volume (Barth et al., 1992), and provide ∼90% of myocardial ATP to support the contraction–relaxation cycle (Tait and Green, 2012). Mitochondria are not only a key source of reactive oxygen species (ROS) but also the main target of ROS damage (Turrens et al., 2003). As a vital intracellular organelle responsible for energy metabolism, loss of mitochondrial integrity and function is a pathological factor for abnormal cardiac structure and cardiac dysfunction after I/R (Yang et al., 2019). Thus, mitochondrial dysfunction seems to be a primary target for therapy after IR. However, despite the development of promising mitochondria-targeting drugs, very few have been used successfully in the clinic. Betaland colleagues indicated that in the future advances in therapy for myocardial IR would come from improvement of the treatments already available rather than the discovery of new drugs (Ibáñez et al., 2015).

Danhong injection (DHI) is composed of Salvia miltiorrhiza Bge (Danshen) and Carthamus tinctorius L (Honghua). Traditional Chinese medicine will receive even more attention in the coming years for its superiority in the prevention and treatment of cardiovascular diseases (Liu and Huang, 2016). DHI is a formulation based on traditional Chinese medicine used widely for the treatment of blood stasis and paralytic stroke. DHI has been approved by the Chinese Food and Drug Administration, and has been included in the emergency-treatment plan of clinical ischemic cardio-cerebrovascular disease (Fu et al., 2018). A study in 30,888 patients in 37 hospitals from six provinces in China showed that the incidence rate of adverse drug reactions using DHI was 3.50% (Li et al., 2015). DHI enhanced the prevalence of total efficacy significantly and reduced the prevalence of major adverse cardiovascular events after PCI for patients with acute coronary syndrome or ST-elevation myocardial infarction (Zhao et al., 2018; Zou et al., 2018; You et al., 2019). Recently, the pharmacologic effects and mechanism of action of DHI in cardiovascular disease have been studied extensively. These effects have included: anti-inflammatory activity *via* nuclear factor-kappa B (NF-κB) (Chen et al., 2019), promotion of angiogenesis *via* a microRNA-126/extracellular signal-regulated kinase/vascular endothelial growth factor pathway (Li et al., 2019): anti-atherosclerotic action *via* ATP-binding cassette transporter 1 (Su et al., 2013); endothelial protection by inhibition of lipopolysaccharide-, oxidized low-density lipoprotein, or cholesterolcrystal-induced activation of NF-κB, c-jun and p38 pathways (Lyu et al., 2017). In addition, DHI has been reported to alleviate mitochondrial swelling in an IR model in minipigs (Ma et al., 2010). However, few reports have shown the underlying mechanism of action of DHI in maintenance of mitochondrial integrity after IR.

Here, DHI was found to serve as a moderator of cellular pathways involved in mitochondrial integrity and to protect cardiomyocytes from apoptosis by maintaining mitochondrial function and then, finally, decreasing the infarct size after IR. The present study highlights a mechanism for increasing cardiomyocyte viability and the potency of DHI as a mitochondrial protectant in treating IR through shutting down pro-apoptotic pathways.

## Materials and Methods

### Identification of Danhong Injection Constituents Using HPLC

DHI was obtained from Heze Buchang Pharmaceutical Co., Ltd. and prepared from aqueous extracts of roots of Salvia miltiorrhiza Bge and flowers of *Carthamus tinctorius* L in the ratio of 3:1. The constituents of DHI were detected by HPLC. Briefly, HPLC analysis was performed on an Agilent 1200 system including a G1311A QuatPump, a G1322A degasser, a G1315D diode array detector, a G1329A ALS with a 20 *µ*L loop. The HPLC column used was Hypersil ODS-C18 (250 × 4.6 mm i.d., 5 *µ*m, Agilent, CA, USA). The mobile phase consisted of water containing 0.1% phosphoric acid (phase A) and acetonitrile (phase B). The gradient program was as follows: 0–6 min, 5% B, 6–16 min, 5–15% B, 16–30 min, 15–26% B, 30–40 min, 26–30% B, 40–55 min, 30–90% B. The flow rate was 1.0 ml/min, and the wavelengths were 280 nm.

### Ischemia/Reperfusion Model and Treatment

Male Sprague-Dawley rats (200–220 g) were purchased from Zhejiang Chinese Medical University. All experiments were approved by the Guide for the Care and Use of Laboratory Animals published by the United States National Institutes of Health (NIH Publication No. 85-23, revised 1996) and approved by the Institutional Animal Care and Use Committee of Zhejiang Chinese Medical University. The animals were fed a standard laboratory diet with free access to food and water and housed under controlled temperature (22 ± 1°C) and humidity (65–70%) with a 12:12 h light : dark cycle. For I/R induction, rats were anesthetized by pentobarbital sodium (50 mg/kg, *i.p*.) and ventilated via tracheal intubation and a rodent ventilator, then, the left anterior descending coronary artery was encircled by an 8-0 nylon suture, reperfusion was executed after myocardial ischemia for 45 min. Low dose DHI treatment (1 ml/kg, *i.v.*, L-DHI), high dose DHI treatment (2 ml/kg, *i.v.*, H-DHI) (Wang et al., 2012) or saline alone (I/R) were administered 7 days before surgical procedure and once after operation.

### Staining With 2,3,5-Triphenyltetrazolium chloride

After rats had been killed, 2% Evans Blue dye (Sigma–Aldrich, Saint Louis, MO, USA) was injected (retrograde) into the ascending aorta to mark the area at risk. Then, the heart was cut into five slices, followed by incubation in 1% 2,3,5-Triphenyltetrazolium chloride solution (Sigma–Aldrich) at 37°C for 10 min to identify the infracted myocardium. The extent of the area of necrosis was quantified on the basis of infarct area/area at risk (INF/AAR) and area at risk/left ventricular (AAR/LV) ratios.

### Immunofluorescence Staining of Tissues

Hearts were dehydrated in 30% sucrose solution, embedded in Tissue-Tek^®^ Optimal Cutting Temperature Compound (Sakura Finetek, Torrance, CA, USA), snap-frozen in liquid nitrogen, and cut into sections of thickness 5 μm for staining. Sections were washed with phosphate-buffered saline, permeabilized with 0.2% Triton X (Sigma–Aldrich) and blocked with 5% bovine serum albumin (Gibco, Billings, MT, USA). Apoptosis was evaluated with a terminal deoxynucleotidyl transferase dUTP nick end labeling (TUNEL) kit (Roche Applied Science, Basel, Switzerland); cell proliferation was incubated with ki67 (Abcam, United Kingdom) and ROSs was stained with dihydroethidium (DHE, Beyotime Biotechnology, China). Cardiomyocytes were stained with troponin I (Abcam, United Kingdom) and nuclei were counterstained with 4′,6-diamidino-2-phenylindole (DAPI; Vector Laboratories, Burlingame, CA, USA).

### Cell Culture

H9C2 cells were purchased from the American Type Culture Collection (Manassas, VT, USA). Passages between 4 and 8 were used for experiments. H9C2 cells were cultured in Dulbecco’s modified Eagle’s medium (DMEM; Gibco) supplemented with 20% fetal bovine serum (FBS; Gibco) and grown in a cell incubator (Thermo Scientific, Waltham, MA, USA) in an atmosphere of 1% O_2_, 94% N_2_, and 5% CO_2_ at 37°C.

### Construction and Infection of Lentivirus

Construction of the recombinant lentivirus with nuclear factor erythroid 2-related factor 2 (Nrf2) was undertaken by Shanghai Taitool Bioscience (Shanghai, China). For infection of H9C2 cells, 10^5^ cells in a six-well plate were infected with lentivirus in the presence of 10 μg/ml of polybrene (Millipore, Bedford, MA, USA) for 12 h, and then the growth medium was replaced. Expression of green fluorescent protein in cells was observed under fluorescence microscopy after 48 h.

### Cell Viability

H9C2 cells (10^4^ cells/well) were cultured on 96-well plates. After 24 h, the medium was replaced with 0.1 ml of DMEM without glucose or FBS, and without or with DHI treatment at a series of concentrations. Then, cells were cultured under hypoxic conditions for 16 h and reoxygenation for 2 h. Cell viability was measured by Cell Counting Kit (CCK)-8 Kit (Dojindo, Tokyo, Japan) with a universal microplate spectrophotometer (SpectraMax M5; Molecular Devices, Silicon Valley, CA, USA).

### Cell Apoptosis

H9C2 cells were cultured in 24-well plates (1 × 10^5^ cells/well) under normal or hypoxic conditions for 16 h and reoxygenation for 2 h, and then apoptotic cells were stained by a terminal deoxynucleotidyl transferase dUTP nick end labeling (TUNEL) kit (Roche Applied Science, Basel, Switzerland), the nuclei were stained with DAPI (Vector Laboratories, Burlingame, CA, USA). The apoptosis rate (%) = The number of TUNEL positive and DAPI positive cells/The number of DAPI positive cells × 100%.

### Transmission Electron Microscopy

Transmission electron microscopy (TEM) was used to measure the length of mitochondria. In brief, 10^6^cells were fixed with 2.5% glutaraldehyde for 12 h. After washing thrice with phosphate-buffered saline, cells were post-fixed with 1% OsO_4_ for 2 h. Next, specimens were dehydrated by ethanol solutions of different concentrations, followed by acetone for overnight infiltration. Furthermore, specimens were embedded in Spurr resin and sectioned using an ultramicrotome (EM UC7; Leica, Wetzlar, Germany). Sections were stained with uranyl acetate and alkaline lead citrate. Images were obtained with a transmission electron microscope (H-7650; Hitachi, Tokyo, Japan).

### Immunofluorescence Staining for Cells

H9C2 cells (2 × 10^4^ cells/well) were cultured on 24-well plates. After 24 h, the medium was replaced with 0.1 ml of DMEM without glucose and FBS, and with or without DHI treatment at a series of concentrations, and then cultured under hypoxic conditions for 16 h and reoxygenation for 2 h. For live cell, MitoTracker^®^ Green FM (Invitrogen Technology, BSN, USA) was used for mitochondrial morphology staining and Calcein-AM (calcein) kit (Life Technologies, Grand Island, NY, USA) was used for mitochondrial permeability transition pore (mPTP) opening detection. For fixed cells by 10% formaldehyde were stained for ROSs by DHE, mitochondrial transmembrane potential by JC-1 (Beyotime Technology, China) and nuclear transcription by primary antibodies against Nrf2 and the corresponding secondary antibodies. The nuclei were stained with DAPI (Vector Laboratories, Burlingame, CA, USA).

### Flow Cytometry

H9C2 cells (2 × 10^5^ cells/well) were cultured on six-well plates. After 24 h, the medium was replaced with 0.1 ml of DMEM without glucose or FBS, and without or with DHI treatment at a series of concentrations, and then cultured under hypoxic conditions for 16 h and reoxygenation for 2 h. Cells werestained by dichloro-dihydro-fluorescein diacetate (DCFH-DA; Beyotime Technology) or MitoSOX^®^ Red (Invitrogen) and detected by flow cytometry with a BD FACS Count II Flow Cytometer (BD Biosciences, San Jose, CA, USA).

### Detection of L-glutathione/Oxidized Glutathione Ratio and Superoxide Dismutase Activity

H9C2 cells (2 × 10^5^ cells/well) were cultured on six-well plates. After 24 h, the medium was replaced with 0.1 ml of DMEM without glucose or FBS, and without or with DHI treatment, and then cultured under hypoxic conditions for 16 h and reoxygenation for 2 h. The ratio of L-glutathione (GSH)/Oxidized Glutathione (GSSG) and the content of Ratio and Superoxide Dismutase (SOD) were measured according to the instructions of the assay kits provided by Beyotime Biotechnology.

### Measurement of ATP Content

H9C2 cells (2 × 10^5^ cells/well) were cultured on six-well plates. After 24 h, the medium was replaced with 0.1 ml of DMEM without glucose or FBS, and without or with DHI treatment at a series of concentrations, and then cultured under hypoxic conditions for 16 h and reoxygenation for 2 h. The ATP level of cells was measured by an ATP kit (Beyotime Technology).

### Detection of Oxygen Consumption

Oxygen consumption in H9C2 cells was measured with a respirometer (Oxygraph-2k; (Oroboros Instruments, Vienna, Austria). Briefly, 5 × 10^5^cells were suspended in respiration media (0.5 mM EGTA, 3 mM MgCl_2_.6H_2_O, 60 mM potassium lactobionate, 20 mM taurine, 10 mM KH_2_PO_4_, 20 mM HEPES, 110 mM sucrose, 1 g/L fatty acid-free bovine serum albumin, pH 7.1) and injected to the chamber by microsyringe. The basal rate of oxygen consumption was detected in the absence of substrates. The maximum rate of oxygen consumption was detected after addition of 4 *µ*M carbonyl cyanide 4-(trifluoromethoxy)-phenylhydrazone (Sigma–Aldrich).

### Real-Time PCR

Total RNA was extracted by Trizol reagent (Invitrogen Technology, BSN, USA). The gene expression was examined by real-time PCR (Thermo fisher scientific, MA, USA) using SYBR Green PCR Master Mix (Takara, Japan). The expression data relative to Normoxia were calculated using 2-ΔΔCT. The gene primers are as follows: Nrf2 forward: 5′-GAC​CTA​AAG​CAC​AG-CCA​ACA​CAT-3′, Reverse: 5′-CTC​AAT​CGG​CTT-GAA​TGT​TTG​TC-3′; keap1 forward: 5′-CCT​GTC​TGT​TGT​CTC​TGC​TTA​C-3′, Reverse: 5′-GAA​GTT​GGG​TCA​TTG​GCT​TCT​A-3′; GAPDH forward: 5′-GCA​CCG​TCA​AGG​CTG​A-GAA​C-3′; Reverse: 5′-ATG​GTG​GTG​AAG​ACG-CCA​GT-3′.

### Western Blot

Cells were lyzed in 2.5× sodium dodecyl sulfate (SDS) gel loading buffer (30 mM Tris-HCl, pH 6.8, 1% SDS, 0.05% bromphenol blue, 12.5% glycerol, and 2.5% mercaptoethanol) and boiled for 30 min; the proteins were prepared for separation on 12% SDS polyacrylamide gels, followed by electro-transferring to polyvinylidene difluoride (PVDF) membranes (Millipore, MA, USA) and incubated with the primary antibodies: rabbit anti-Bcl-2 (1:1,000, ab32124,Abcam, United Kingdom), rabbit anti-Bax (1:1,000, ab32503, Abcam, United Kingdom), rabbit anti-cleaved caspase3 (1:1,000, ab49822, Abcam, United Kingdom), rabbit anti-cytochrome c (1:1,000, 4272s, CST, MA, USA),rabbit anti-JNK1 + JNK2 + JNK3 (phosphor T183 + T183 + T221) (1:1000, ab124956,Abcam, United Kingdom), rabbit anti-JNK1 + JNK2 + JNK3 (1:1,000, ab179461,Abcam, United Kingdom), rabbit anti-Keap1 (1:1,000, ab139729, Abcam, United Kingdom), Nrf2 (1:1,000, ab31163,Abcam) and mouse anti-β-actin (1:3,000, KC-5A08, Kangcheng, China). Protein bands were visualized after horseradish peroxidase-conjugated secondary antibodies incubation and detected by a chemiluminescence ECL Western-blotting system (Millipore, MA, USA).

### Statistical Analysis

Data are presented as the mean ± standard deviation. Statistical significance was determined using one-way ANOVA for comparisons among more than two groups and one-tailed t-tests for comparisons between two groups. *p* value less than 0.05 was considered statistically significant.

## Results

### Components of Danhong Injection as Identified by High-Performance Liquid Chromatography

**FIGURE 1:**
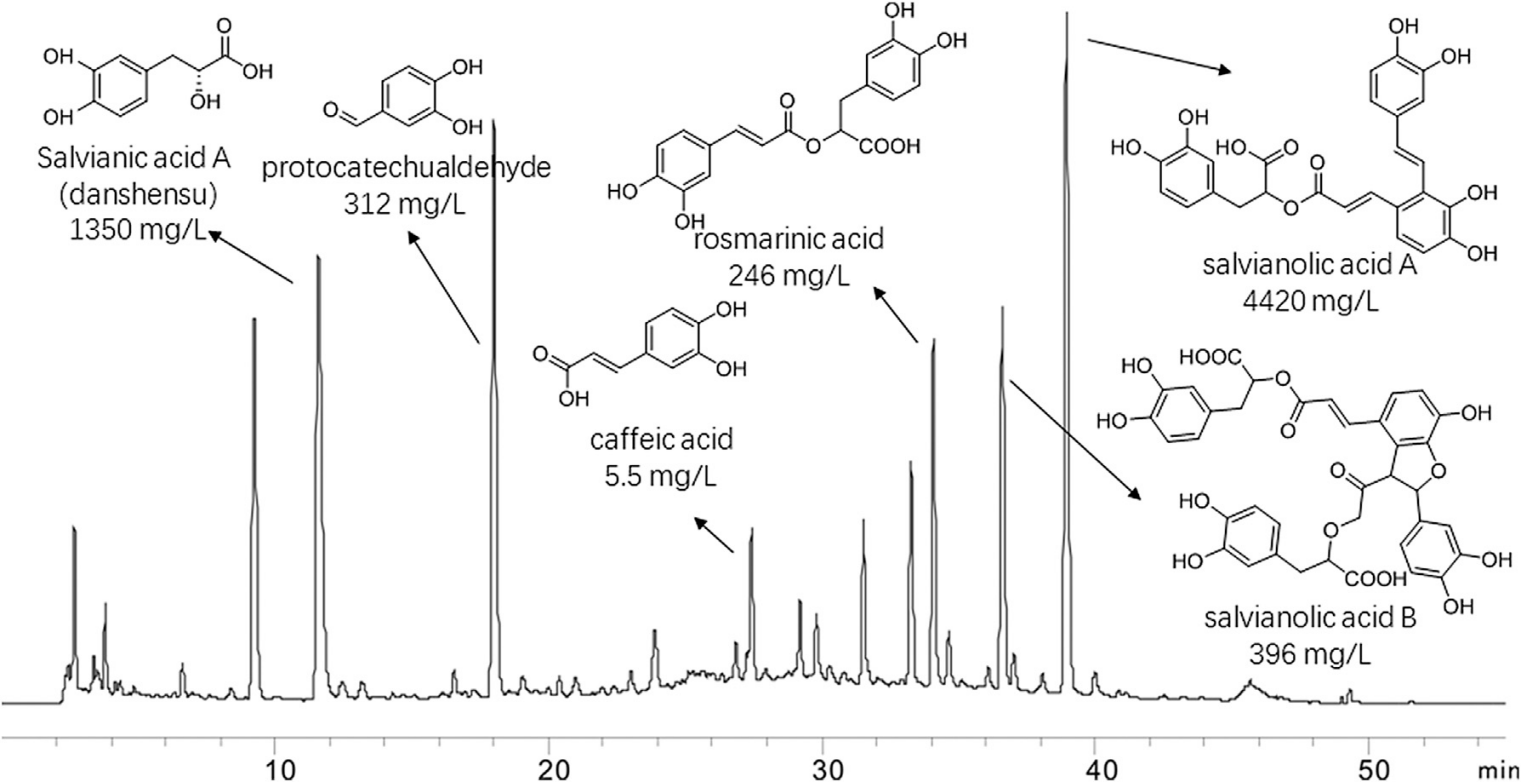
Chromatogram of danhong injection by HPLC analysis at 280 nm. Thestructures and their relative contents of six representive compounds were shown.

The major compounds and their relative content in DHI were identified: Salvianic acid A (danshensu) (1,350 mg/L), protocatechualdehyde (312 mg/L), rosmarinic acid (246 mg/L), caffeic acid (5.5 mg/L), salvianolic acid A (4420 mg/L) and salvianolic acid B (396 mg/L). The chromatogram, structures and relative content of the major compounds of DHI are shown in Figure 1.

### Danhong Injection Attenuates the Size of the Myocardial Infarction Rat Hearts After Ischemia/Reperfusion

**FIGURE 2:**
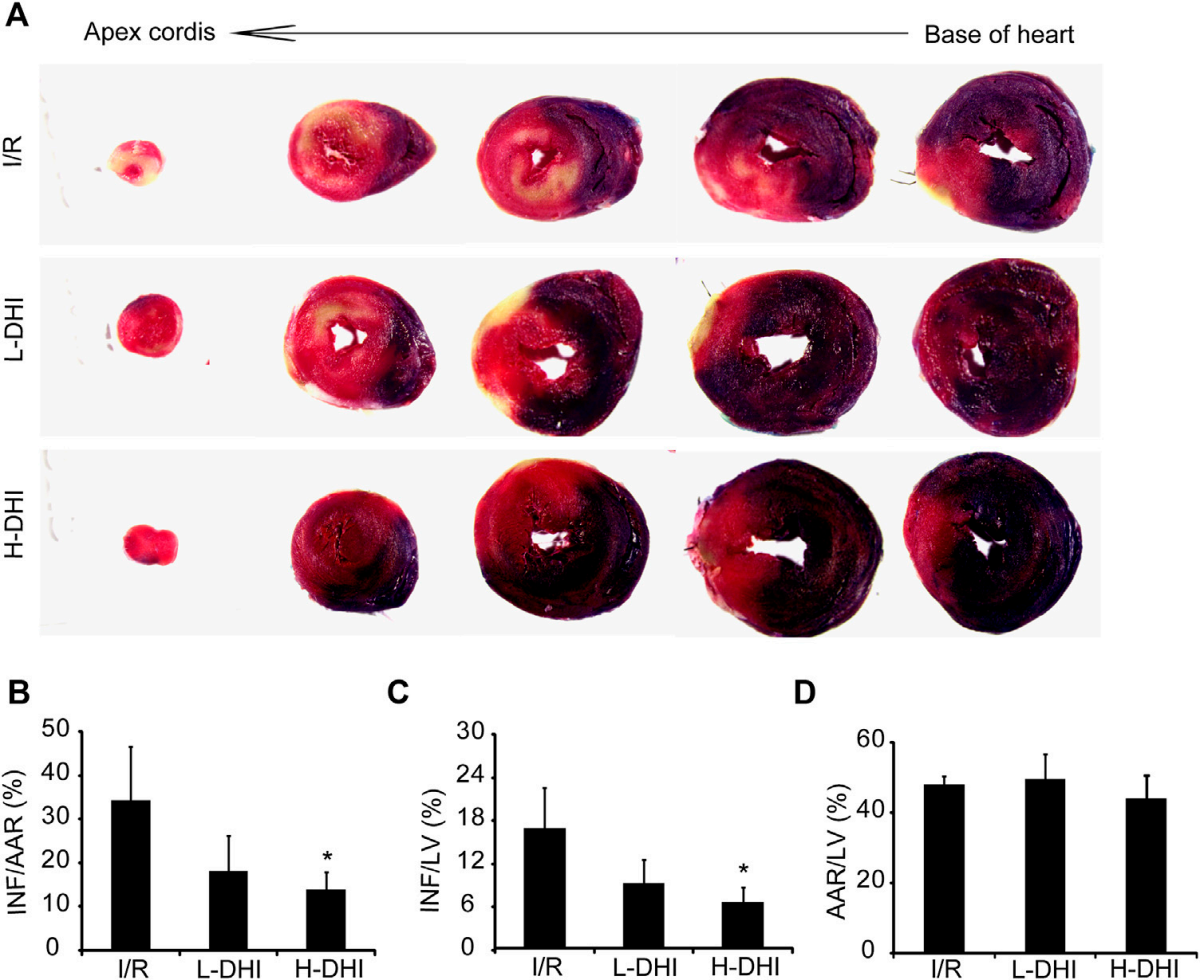
Measures of infarct size in rat hearts after ischemia/reperfusion (I/R) injury is better after treatment with danhong injection (DHI) than with no treatment. **(A)** 24 h after I/R and treatment, the heart was cut into five layers from base of heart to apexcordis, sections of the left ventricles (LV) were stained to determine regions of normal (blue), infarcted area (white) and area at risk (red). **(B–D)** Infarct area/area at risk (INF/AAR), the risk/left ventricular (INF/LV) and area at risk/left ventricular (AAR/LV) ratio was quantified, n (number of animals) ≥4, *p < 0.05 vs. I/R group.

The therapeutic effect of DHI on I/R *in vivo* was evaluated. An IR model was induced surgically in rat hearts. Then, animals were treated with DHI at different doses. The INF/AAR ratio and INF/LV ratio in the IRI group was 34.4 and 16.5%, respectively. Treatment with a high dose of DHI decreased the INF/AAR ratio and INF/LV ratio significantly to 14.1 and 6.3%, respectively. Treatment with a low dose of DHI could reduce the INF/AAR ratio and INF/LV ratio as compared with that in the I/R group, but not significantly (Figures 2A,C). The AAR/LV ratio was not significantly different among these groups, which suggested that the same ischemic area was present in the three groups (Figure 2D).

### Danhong Injection Improves Cardiomyocyte Survival and Suppresses Oxidative Stress After Ischemia/Reperfusion

**FIGURE 3:**
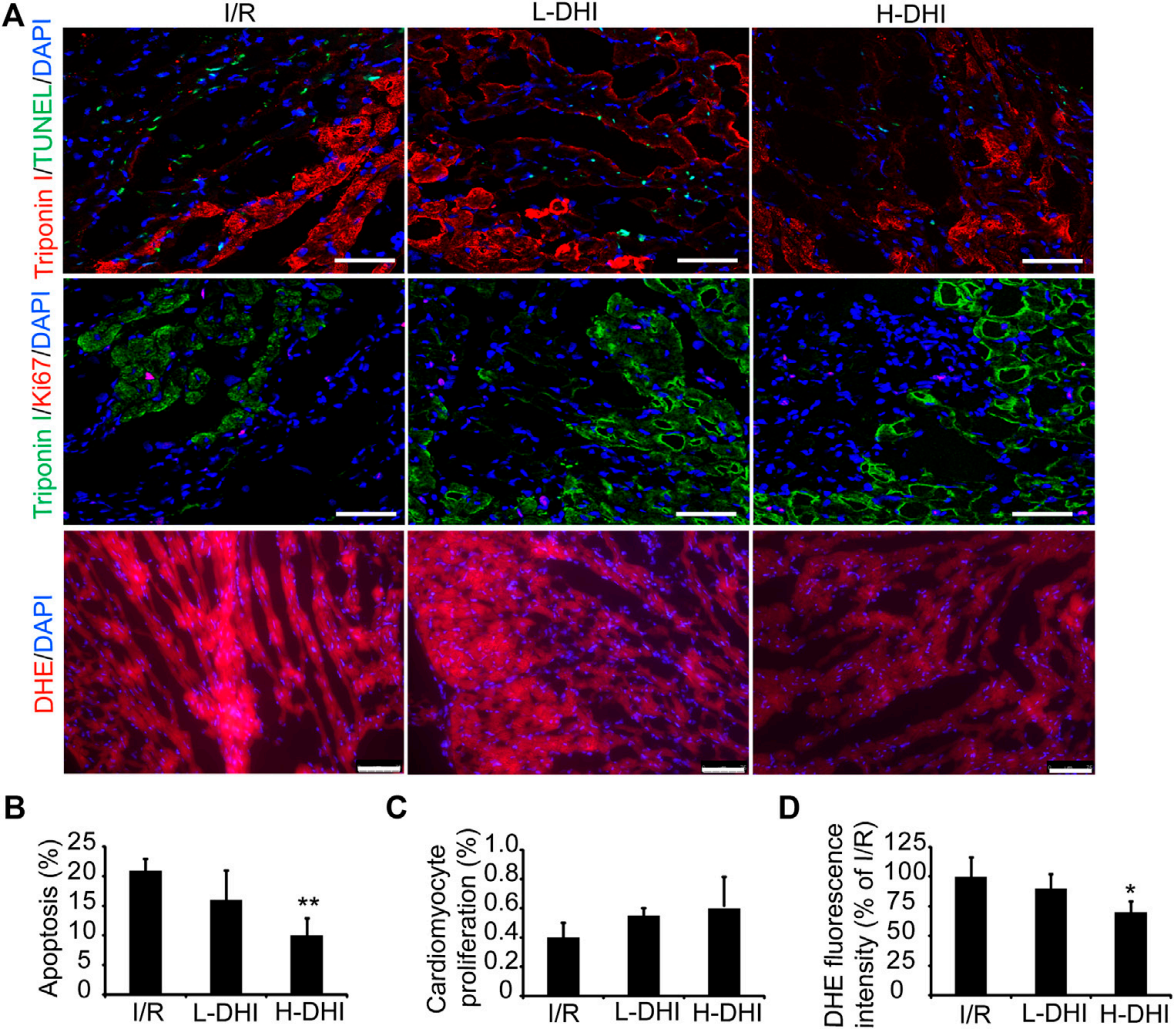
Danhong injection (DHI) improves reparative potency of DHI for myocardium in rats after ischemia/reperfusion (I/R). **(A)** Sections collected from the border zones of infarction were stained for TUNEL (green, bar = 100 μm) to identify apoptotic cells, Ki67 (red, bar = 100 μm) to identify cell proliferation and DHE (red, bar = 75 μm) to identify reactive oxygen species generation. **(B)** Apoptosis was quantified as the percentage of TUNEL-positive cells. Cardiomyocytes were visualized via staining for troponin I (red), and nuclei were counterstained with DAPI (blue). **(C)** Cardiomyocyte proliferation was quantified as the percentage of Ki67 and Troponin I-positive cells (green), and nuclei were counterstained with DAPI (blue). **(D)** DHE fluorescence intensity was quantified. n (number of animals) = 4,*p < 0.05 or**p < 0.01 vs. I/R group.

To determine if DHI could protect cardiomyocytes from the apoptosis induced by I/R, apoptotic cells were detected by co-staining of troponin I and TUNEL.A high dose of DHI decreased the apoptosis rate significantly as compared with that in the I/R group, but there was no significant difference in cardiomyocyte proliferation among I/R, high-dose DHI or low-dose DHI groups (Figures 3A–C). DHI treatment (especially the high-dose group) could obviously inhibit ROS generation according to DHE staining (Figures 3A,D).

### Danhong Injection Reduces Apoptosis of H9C2 Cells After Hypoxia/Reoxygenation Injury

**FIGURE 4:**
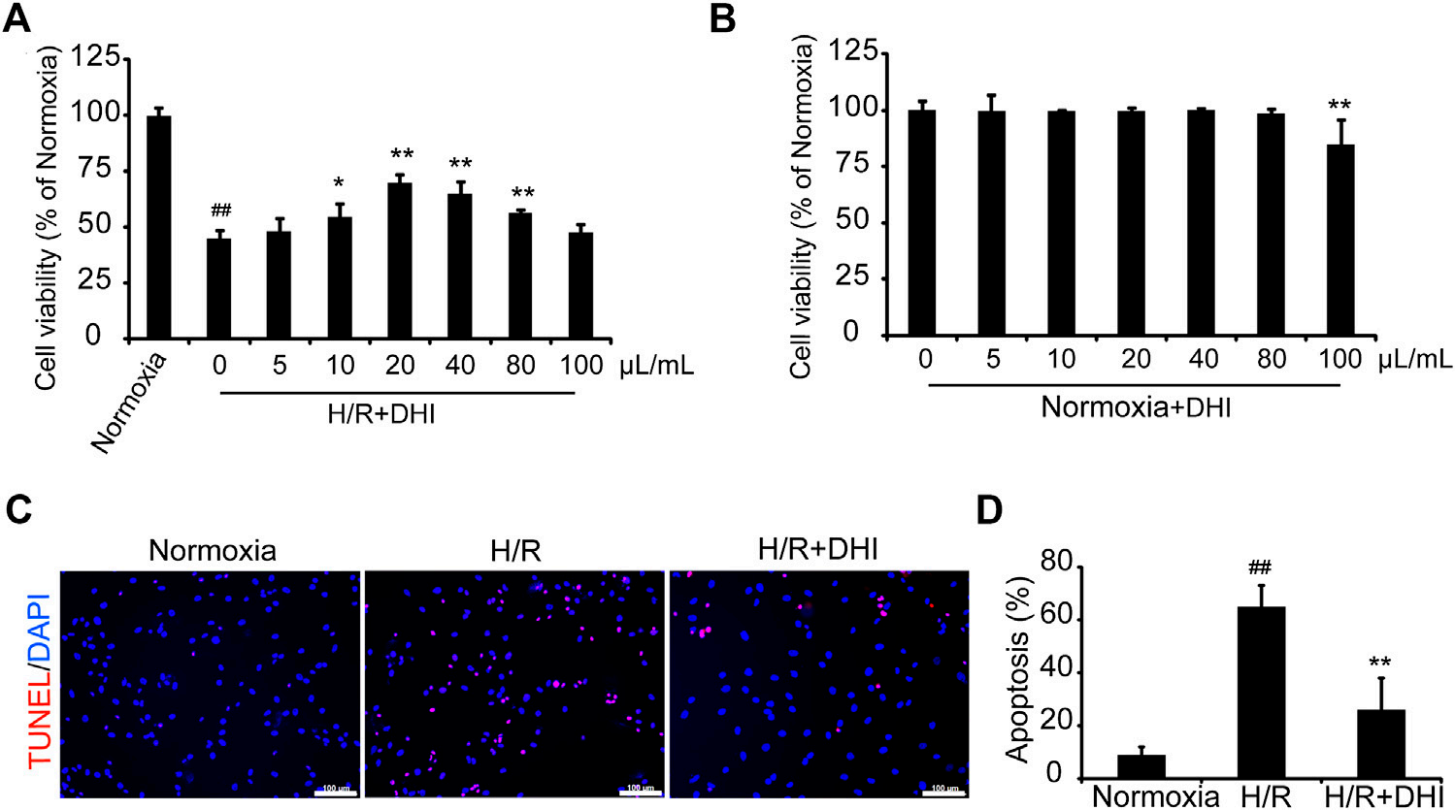
Danhong injection (DHI) protects H9C2 from hypoxia-reoxygenation induced apoptosis. **(A,B)** H9C2 were plated on a 96-well plate with or without hypoxia/reoxygenation (H/R) and DHI treatment, the cell viability was evaluated via optical density (OD) with a CCK-8 kit, n = 4.*p < 0.05or**p < 0.01 vs. H/R group or Normoxia + DHI (0 μL/ml); ^##^ *p* < 0.01 vs. Normoxia. **(C)** TUNEL stained, and imaged via fluorescence microscopy (red: TUNEL, blue: DAPI-stained nuclei, bar = 100 *µ*m); then, **(D)** Apoptosis was quantified as the proportion of positively stained cells, n = 3. **p < 0.01 vs. H/R group; ^##^ *p* < 0.01 vs. Normoxia.

CCK-8 was used to measure cell viability with or without a series of DHI concentrations under normoxia or hypoxia condition. DHI (10–80 μL/ml) treatment increased cell viability significantly as compared with that in the hypoxia/reoxygenation (H/R) group (Figure 4A), and there was no significantly change in cell viability among DHI (0–80 μL/ml) treatment groups under normoxia condition (Figure 4B). Hence, we chose an optimal dose of 20 μL/ml of DHI for subsequent experiments. To ascertain if the cytoprotective effect of DHI observed *in vitro* may improve the survival of H9C2 cells, the TUNEL assay was used to detect their apoptosis after H/R. The apoptosis rate was lower in the DHI group (26%) than that in the H/R group (65%) (Figures 4C,D).

### Danhong Injection Scavenges Free Radicals After Hypoxia/Reoxygenation

**FIGURE 5:**
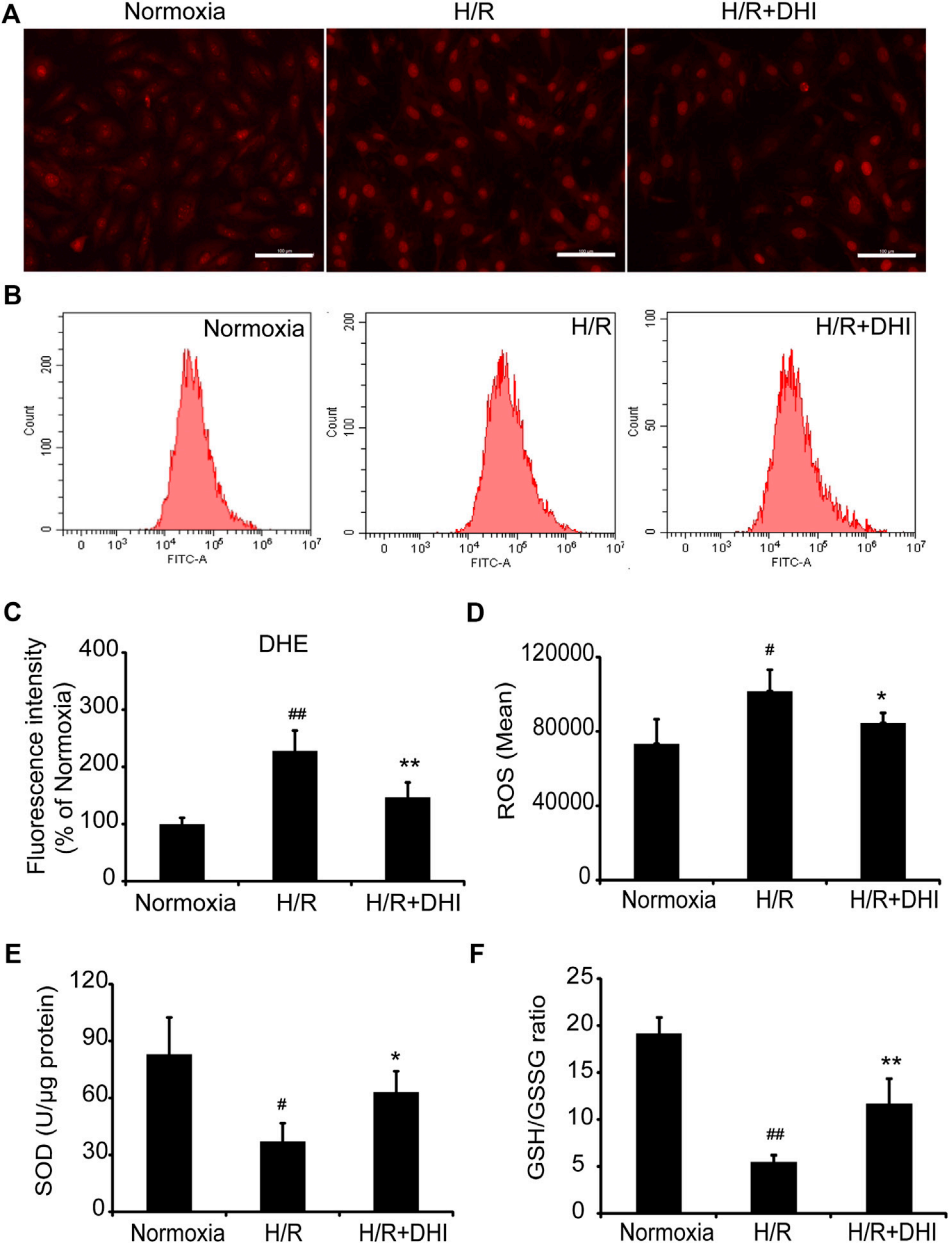
Danhong injection (DHI) inhibits oxidative stress after hypoxia/reoxygenation (H/R) injury. reactive oxygen species generation was detected and quantified in Normoxia, H/R and H/R + DHI group by **(A,C)** DHE staining (bar = 100 μm, n = 3) and **(B,D)** DCFH-DA FACS (n = 3). **(E,F)** The L-glutathione/Oxidized Glutathione ratio and the level of Superoxide Dismutase were detected by kits in Normoxia, H/R and H/R + DHI group, n = 3. *p < 0.05or**p < 0.01 vs. H/R group; ^#^ *p* < 0.05 or ^##^ *p* < 0.01 vs. Normoxia.

During myocardial I/R, formation of oxygen free radicals increases and the scavenging system is limited. Oxidative stress plays a vital part in mitochondrial function and apoptosis. ROS generation was increased significantly in the H/R group as compared with that in the normoxia group, whereas DHI treatment decreased the ROS level significantly according to DHE staining and the DCFH-DA assay (Figures 5A–D). Moreover, the level of biomarkers related to oxidative stress (GSH and SOD) was also measured. The GSH/GSSG ratio and the level of SOD were decreased significantly in the H/R group as compared with that in the normoxia group, and DHI treatment reversed these changes (Figures 5E,F).

### Danhong Injection Maintains the Integrity and Improves the Function of Mitochondria After Hypoxia/Reoxygenation

**FIGURE 6:**
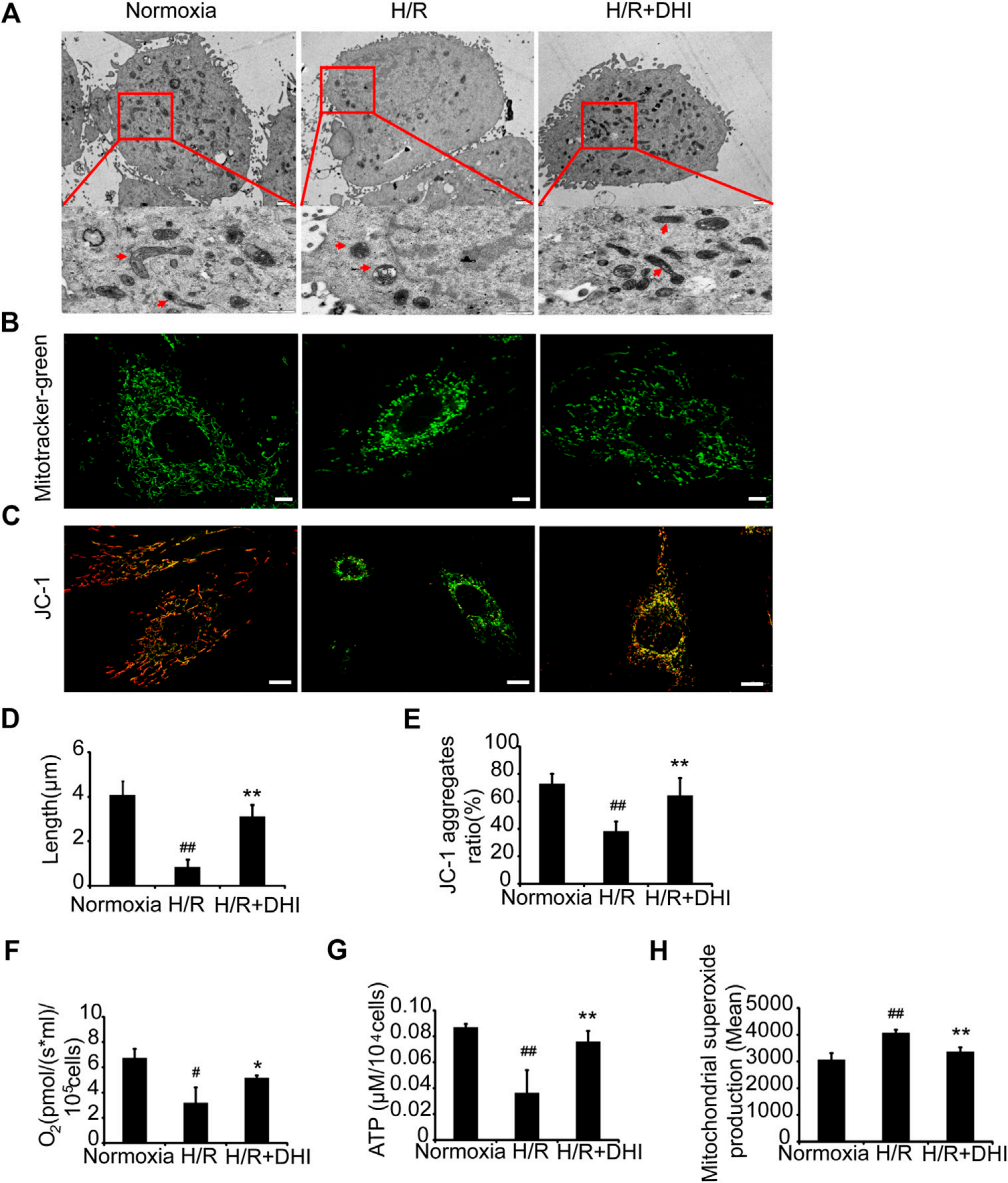
Danhong injection (DHI) maintains mitochondrial integrity and improves mitochondrial function of H9C2 after hypoxia/reoxygenation (H/R) injury. **(A)** Mitochondrial length was detected by TEM (upper: bar = 1 μm, down: bar = 500 nm). **(B)** Mitochondrial integrity was measured by MitoTracker^®^ Green FM (bar = 5 μm). **(C)** Mitochondrial membrane potentials were evaluated via JC-1 staining (Aggregates: red; Monomers: green, bar = 10 *µ*m). **(D)** Mitochondrial length was quantified at least five cells for each group. **(E)** Mitochondrial membrane potentials were quantified in Normoxia, H/R and H/R + DHI group, n = 3. **(F)** Maximum oxygen consumption rates were detected in Normoxia, H/R and H/R + DHI group, n = 3. **(G)** Intracellular ATP levels were measured in Normoxia, H/R and H/R + DHI group, n = 3. **(H)** reactive oxygen species generation by mitochondria was detected and quantified by MitoSOX FACS, n = 3. *p < 0.05or **p < 0.01 vs. H/R group; ^#^ *p* < 0.05or ^##^ *p* < 0.01 vs. Normoxia.

To determine whether DHI affected mitochondrial integrity after exposure to H/R, TEM was used to measure mitochondrial length. The latter was significantly shorter after exposure to H/R than that under normoxia, whereas the mitochondrial length was longer in the DHI group than that in the H/R group (Figures 6A,D). Accordingly, the changes in mitochondrial integrity induced by H/R resulted in significant decreases in the DHI group according to MitoTracker staining (Figure 6B). Mitochondrial function also determines the fate of apoptosis. The mitochondrial membrane potential (MMP) was measured by JC-1 staining. DHI treatment reversed the MMP significantly after H/R (Figures 6C,E). Accordingly, H/R resulted in a significant decrease in the rate of oxygen consumption and ATP level, but these changes were much lower in the DHI group than those in the H/R group (Figures 6F,G). The ROS level produced by mitochondria was increased significantly in the H/R group, and DHI treatment decreased ROS generation significantly (Figure 6H). These findings suggested that DHI decreased ROS generation and, hence, improved mitochondrial function.

### Possible Role for Keap1/Nuclear Factor Erythroid 2-Related Factor 2/JNK in Danhong Injection-Mediated Mitochondrial Integrity and Inhibition of Apoptosis

**FIGURE 7:**
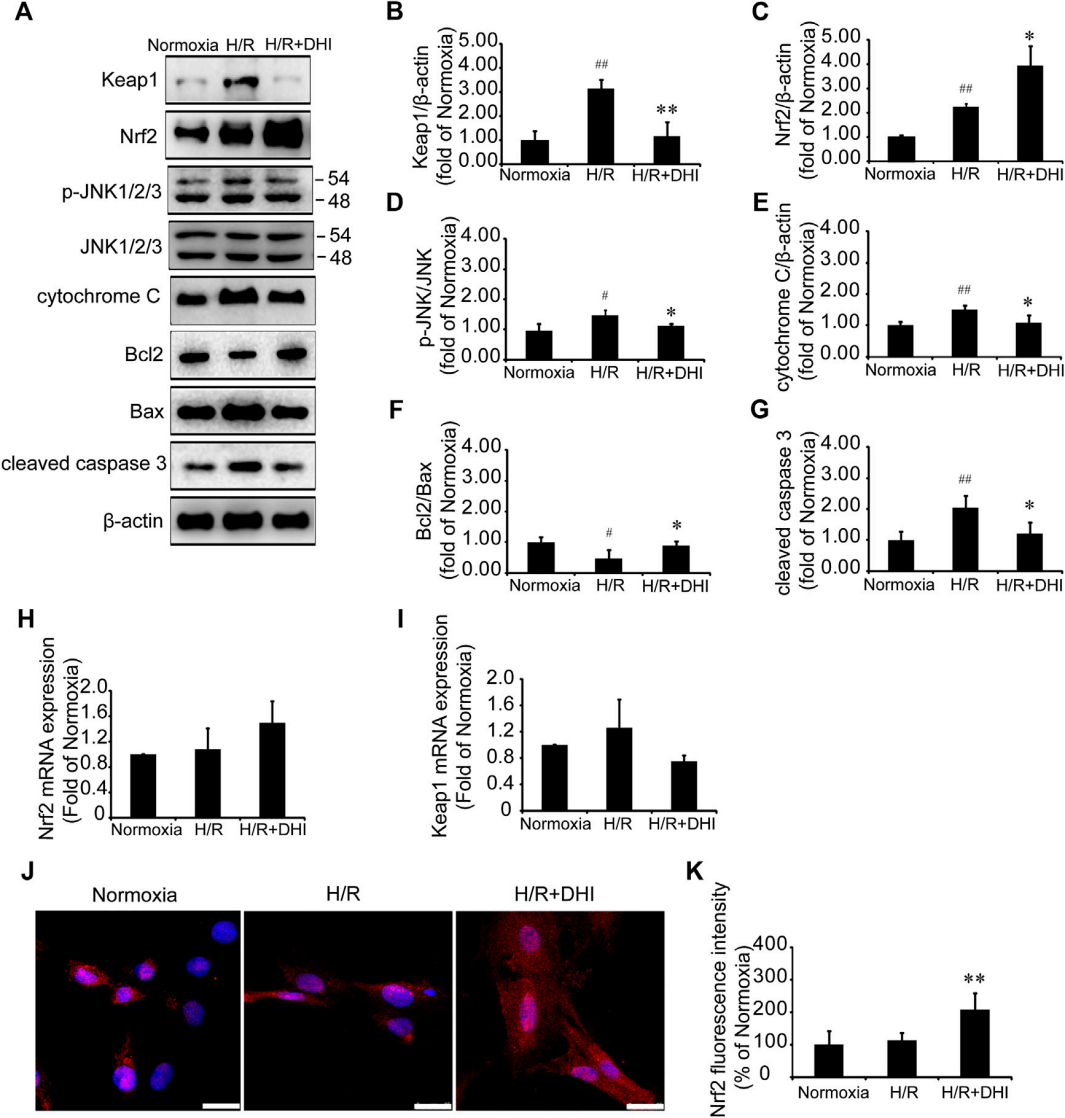
Danhong injection (DHI) mediated mitochondrial integrity and inhibited cell apoptosis through Keap1/nuclear factor erythroid 2-related factor 2 (Nrf2)/JNK pathway. **(A–G)** Keap1, Nrf2, p-JNK/JNK, cytochrome c, Bcl2, Bax, and cleaved caspase 3 protein levels were evaluated and quantified via Western blot in Normoxia, hypoxia/reoxygenation (H/R) and H/R + DHI group, β-actin levels were evaluated to confirm equal loading, n = 3.*p < 0.05or **p < 0.01 vs. H/R group; ^#^ *p* < 0.05 or ^##^ *p* < 0.01 vs. Normoxia. **(H,I)** Keap1and Nrf2 mRNA expressions were detected by quantitative real-time PCR, n = 3. **(J, K)** Nuclear transcription of Nrf2 was measured and quantified by Immunofluorescence staining, bar = 25 μm, n = 3, **p < 0.01 vs. H/R group.

Nrf2 and its downstream molecule JNK participate in cardiomyocyte apoptosis after H/R (Li et al., 2017). Nrf2/JNK is also involved in the regulation of mitochondrial function (Song et al., 2019). Expression of Nrf2 protein and p-JNK protein was increased significantly after H/R, and DHI treatment increased Nrf2 expression significantly (Figures 7A,C) and decreased phosphorylation of its downstream protein (JNK) (Figures 7A,D), but did not change expression of Nrf2 mRNA (Figure 7H). Keap1 is the anchor protein of Nrf2. After I/R, Keap1 expression was increased significantly in the H/R group, which combined with Nrf2 and made it inactive. DHI treatment decreased Keap1 expression significantly (Figures 7A,B) and released Nrf2 to enable translocation to the nucleus (Figures 7J,K), but it did not alter expression of Keap1 mRNA (Figure 7I). H/R also decreased the Bcl2/Bax ratio, increased the level of cytochrome c and activated caspase 3, but this did not occur in the DHI group (Figures 7A,E–G). Collectively, these observations suggested that DHI degraded Keap1 and released Nrf2 to enable translocation to the nucleus, then inhibited JNK phosphorylation to maintain mitochondrial integrity and protect cardiomyocytes from H/R-induced apoptosis.

### Nuclear Factor Erythroid 2-Related Factor 2 Deficiency Abolished the Protective Effects of Danhong Injection on H9C2 Cells Under Hypoxia/Reoxygenation Stimuli

**FIGURE 8:**
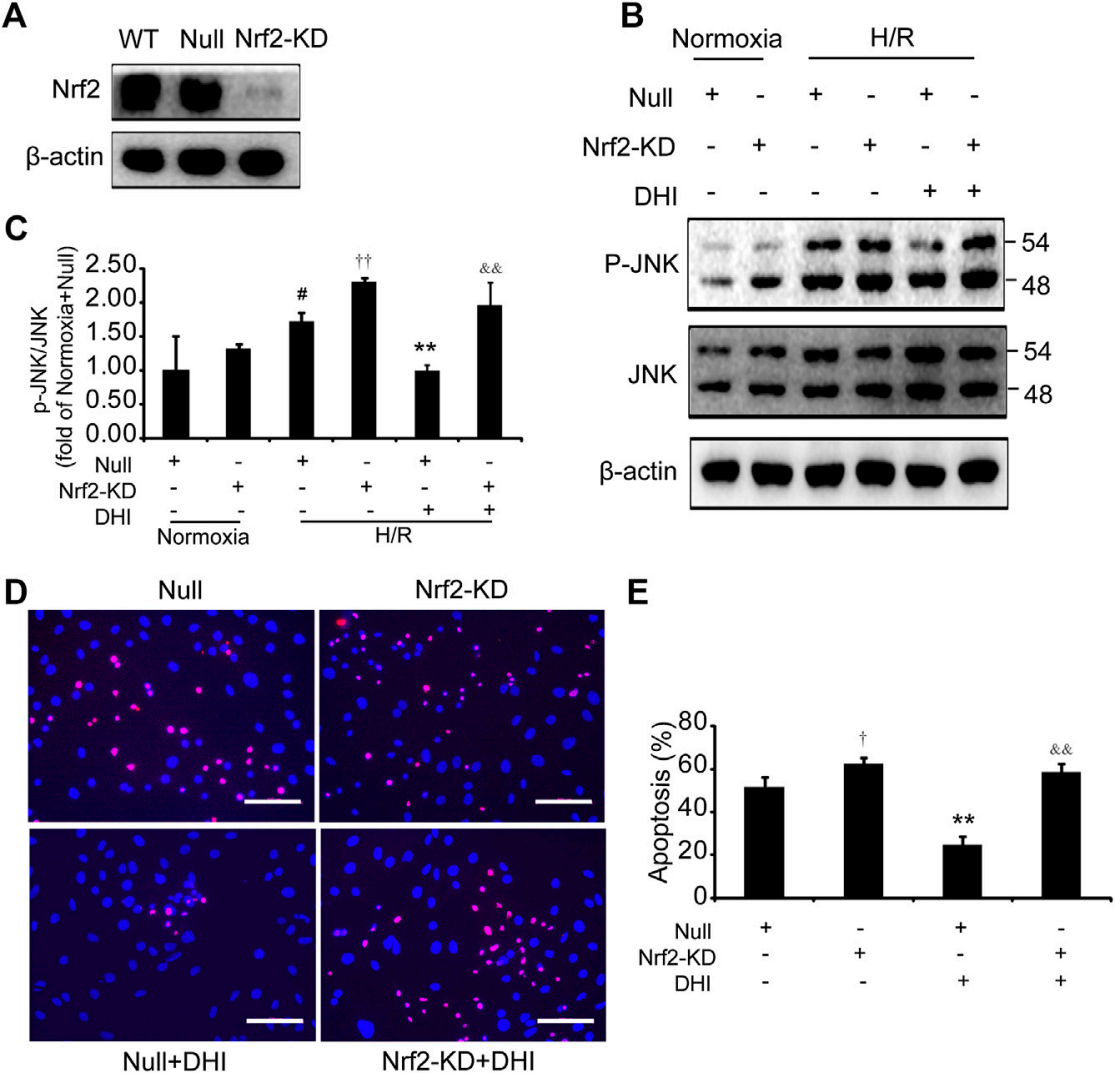
Nuclear factor erythroid 2-related factor 2 (Nrf2) is required in the effect of Danhong injection (DHI) in inhibiting cell apoptosis. **(A)** Nrf2 protein levels were evaluated via Western blot in wild-type H9C2 (WT), in H9C2 transfected with empty vector (Null) and in H9C2 transfected with Nrf2 shRNA (Nrf2-KD); actin levels were evaluated to confirm equal loading. **(B,C)** p-JNK and JNK protein levels were evaluated and quantified via Western blot in H9C2 transfected with empty vector (Null) and in H9C2 transfected with Nrf2 shRNA (Nrf2-KD) under normoxia and hypoxia-reoxygenation (H/R) conditions or in the absence and presence of DHI; actin levels were evaluated to confirm equal loading, n = 3. **(D,E)** H9C2 that had been transfected with empty vector (Null) and Nrf2 shRNA (Nrf2-KD) were stimulated with H/R; then, apoptotic cells were identified *via* TUNEL (red); nuclei were counterstained with DAPI (blue) (bar = 100 *µ*m) and quantified as the percentage of the total number of cells (n = 3), *p < 0.05 or **p < 0.01 vs. H/R + Null or H/R + Null + DHI; ^#^ *p* < 0.05versusNormoxia + Null.

We wished to ascertain if Nrf2 is necessary for the effect of DHI on cell survival and whether JNK is downstream of Nrf2. Hence, we analyzed apoptosis and JNK phosphorylation in H9C2 cells with knockdown of Nrf2 expression (Nrf2-KD) (Figure 8A). Under normoxia, the p-JNK/JNK ratio was increased slightly in H9C2 cells with Nrf2-KD as compared with H9C2 cells transfected with empty vectors (null). After exposure to H/R, a decrease in the p-JNK/JNK ratio was observed in the DHI group as compared with that in the H/R-only group. However, upon Nrf2-KD, the decrease in JNK phosphorylation by DHI was abolished (Figures 8B,C). The decreased apoptosis mediated by DHI was reversed when H9C2 cells were transfected with Nrf2 short hairpin (sh)RNA (Figures 8D,E). These findings suggested that JNK was downstream of Nrf2, and that Nrf2 might be required for DHI-mediated improvements in survival of H9C2 cells after H/R stimulation.

### Nuclear Factor Erythroid 2-Related Factor 2 Is Required for the Danhong Injection-Mediated Improvement in the Integrity and Function of Mitochondria

**FIGURE 9:**
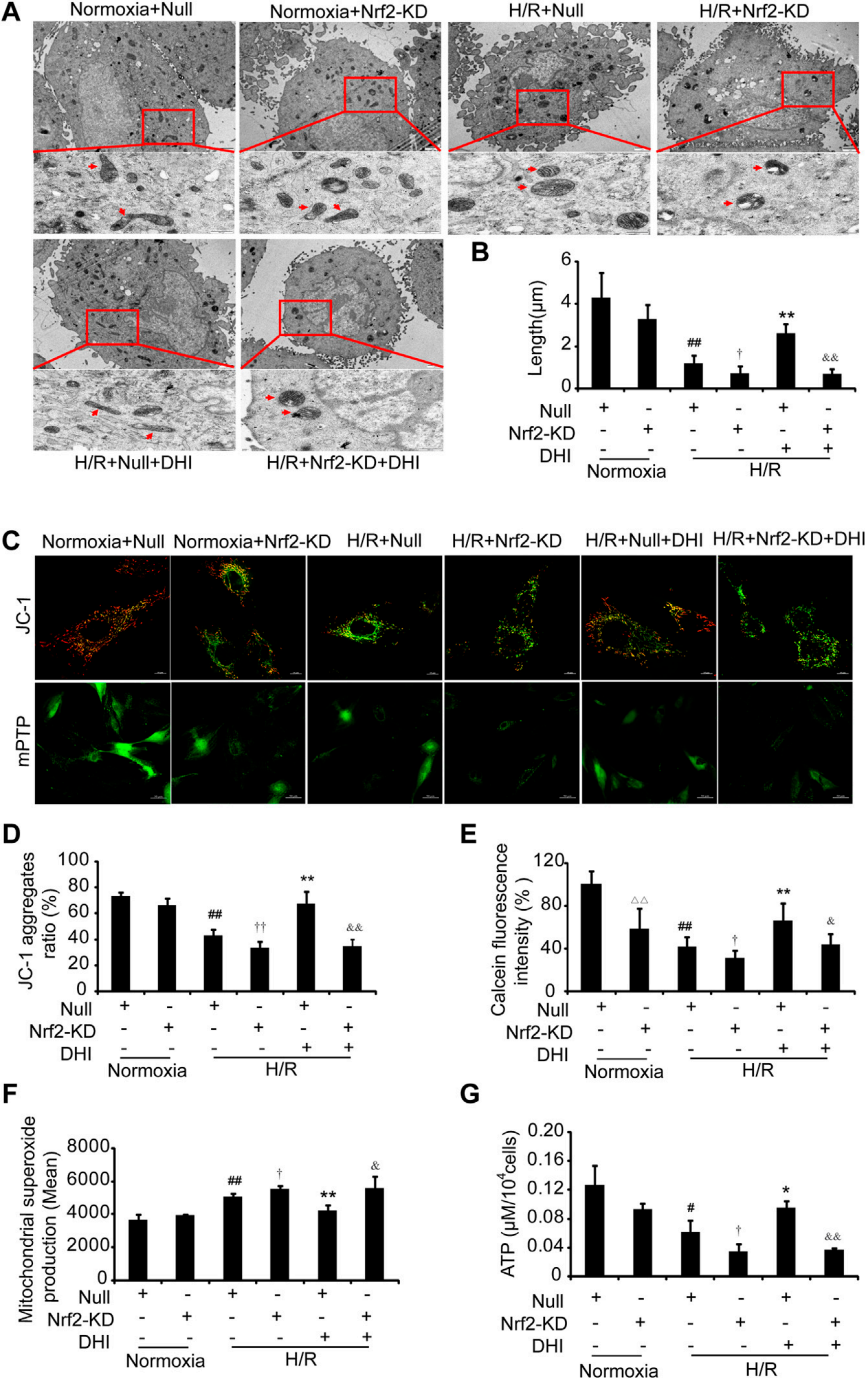
Nuclear factor erythroid 2-related factor 2 (Nrf2) is crucial in Danhong injection (DHI)-mediated mitochondrial integrity. (**A)** Mitochondrial ultrastructures were detected by TEM to show the mitochondrial length (upper: bar = 1 μm, down: bar = 500 nm). **(B)** The mitochondrial length was measured at least five cells for each group. **(C)** JC-1 was used to stain mitochondrial membrane potentials and calcein fluorescence was used to detect the mitochondrial permeability transition pore (mPTP) opening, **(D)** JC-1 aggregates ratio was quantified (Aggregates: red; Monomers: green, bar = 10 *µ*m), n = 3. **(E)** mPTP opening with decreased green fluorescence and calcein fluorescence intensity (% relative to Normoxia + Null) was quantified. **(F)** reactive oxygen species production in mitochondria was detected by FACS and quantified in Normoxia + Null, Normoxia + Nrf2-KD, H/R + Null, H/R + Nrf2-KD, H/R + Null + DHI, and H/R + Nrf2-KD + DHI, n = 3. **(G)** Intracellular ATP levels were evaluated in Normoxia + Null, Normoxia + Nrf2-KD, H/R + Null, H/R + Nrf2-KD, H/R + Null + DHI, and H/R + Nrf2-KD + DHI, n = 3. ^△△^ *p* < 0.01, Normoxia + Nrf2-KD vs. Normoxia + Null; ^#^ *p* < 0.05 or ^##^ *p* < 0.01, H/R + Null vs. Normoxia + Null; ^†^ *p* < 0.05 or ^††^ *p* < 0.01, H/R + Null vs. H/R + Nrf2-KD; *p < 0.05 or **p < 0.01, H/R + Null + DHI vs. H/R + Null; ^&^ *p* < 0.05 or ^&&^ *p* < 0.01, H/R + Null + DHI vs. H/R + Nrf2-KD + DHI.

We wished to ascertain if Nrf2 is necessary for DHI to maintain the integrity and function of mitochondria. Hence, we analyzed the mitochondrial length, MMP, mPTP opening, ATP content, and ROS formation in H9C2 cells with Nrf2-KD. An increase in mitochondrial length was detected in the DHI group compared with that in the H/R-only group, and the protective effect of DHI on mitochondrial length was abolished when Nrf2 was knocked down using Nrf2 shRNA (Figures 9A,B). Furthermore, the increased MMP (Figures 9C,D), reduced mPTP opening (Figures 9C,E), decreased expression of ROS (Figure 9F) and increased ATP production (Figure 9G) mediated by DHI were reversed when H9C2 cells were transfected with Nrf2 shRNA. These findings suggested that Nrf2 was required for DHI-mediated improvements in mitochondrial integrity after H/R stimulation.

## Discussion

We demonstrated that DHI plays a key part in maintenance of mitochondrial integrity and inhibits cardiomyocyte apoptosis and, thus, DHI can protect against I/R. Our results suggest a pathway wherein DHI degrades Keap1 and activates Nrf2 to mediate the downstream protein JNK to maintain the integrity and function of mitochondria. Thus, our results underscore a signaling pathway of Keap1/Nrf2/JNK in regulating mitochondrial homeostasis and inhibiting apoptosis.

The integrity and function of mitochondria are important for cell survival and have a central role in reperfusion-injury pathways (Patil et al., 2015). First, mitochondria are morphologically dynamic organelles regulated by fusion and fission. During apoptosis, mitochondrial fragments shrink (Wallace et al., 1999). Second, in health, mitochondria produce most cellular energy *via* oxidative phosphorylation. In response to oxidative stress after I/R, mitochondria alter the redox potential, release cytochrome c and activate the caspase pathway to trigger apoptosis (Wallace et al., 2005; Guo et al., 2020). Recently, mitochondrial dysfunction has been reported to trigger an integrated stress response in the cytosol (Cadenas et al., 2018). The outcome of reperfusion following myocardial ischemia is worsened if the endogenous ROS produced by mitochondria are increased or if mitochondrial function is impaired. This hypothesis was confirmed in our study because H/R increased ROS production and induced abnormal dynamicsand dysfunction in mitochondria to activate cytochrome c and cleaved caspase three then, finally, triggered cardiomyocyte apoptosis. Studies have suggested that pathways associated with antioxidants and improvement in mitochondrial integrity are important during reperfusion (Kubben et al., 2016), especially for the heart (the organ most reliant on mitochondrial bioenergetics). DHI exerted robust antioxidant and mitochondrial-targeting functions by decreasing ROS levels and maintaining mitochondrial integrity.

Nrf2 is a member of the NF-E2 family. It is a nuclear basic leucine zipper protein that binds to the antioxidant responsive element and activates endogenous antioxidant enzymes in response to oxidative stress (Bae et al., 2013; Liu et al.,. 2020). Its activation is controlled primarily by Keap1. Usually, inactive Nrf2 is coupled with Keap1 and anchored to the cytoskeleton and finally degrades through ubiquitination (Ding et al., 2016) or p62-dependent autophagy (Song et al., 2019). After I/R, expression of Keap1 protein increased, and intracellular expression of free Nrf2 protein decreased (Li et al., 2017). Nrf2 is the key target for I/R in two main ways. First, oxidative stress is mediated by nuclear transcription of Nrf2 and binding to antioxidant responsive elements such as heme oxygenase-1, glutathione reductase, glutathione peroxidase, glutathione S-transferase, and catalase. Second, attenuation of mitochondrial dysfunction is regulated by the Nrf2/JNK pathway (Song et al., 2019) which is also involved in cardiomyocyte death after H/R (Li et al., 2019). Our results suggested that DHI regulated mitochondrial integrity through degrading Keap1 protein expression and increasing Nrf2 protein expression rather than gene levels of Keap1 and Nrf2, then subsequently inhibited JNK phosphorylation in H/R-stimulated cells. DHI mediated mitochondrial integrity in an Nrf2-dependent manner driven by inhibiting JNK phosphorylation as the downstream target because mitochondrial protection was eliminated by Nrf2-KD in H9C2 cells.

## Conclusion

**FIGURE 10:**
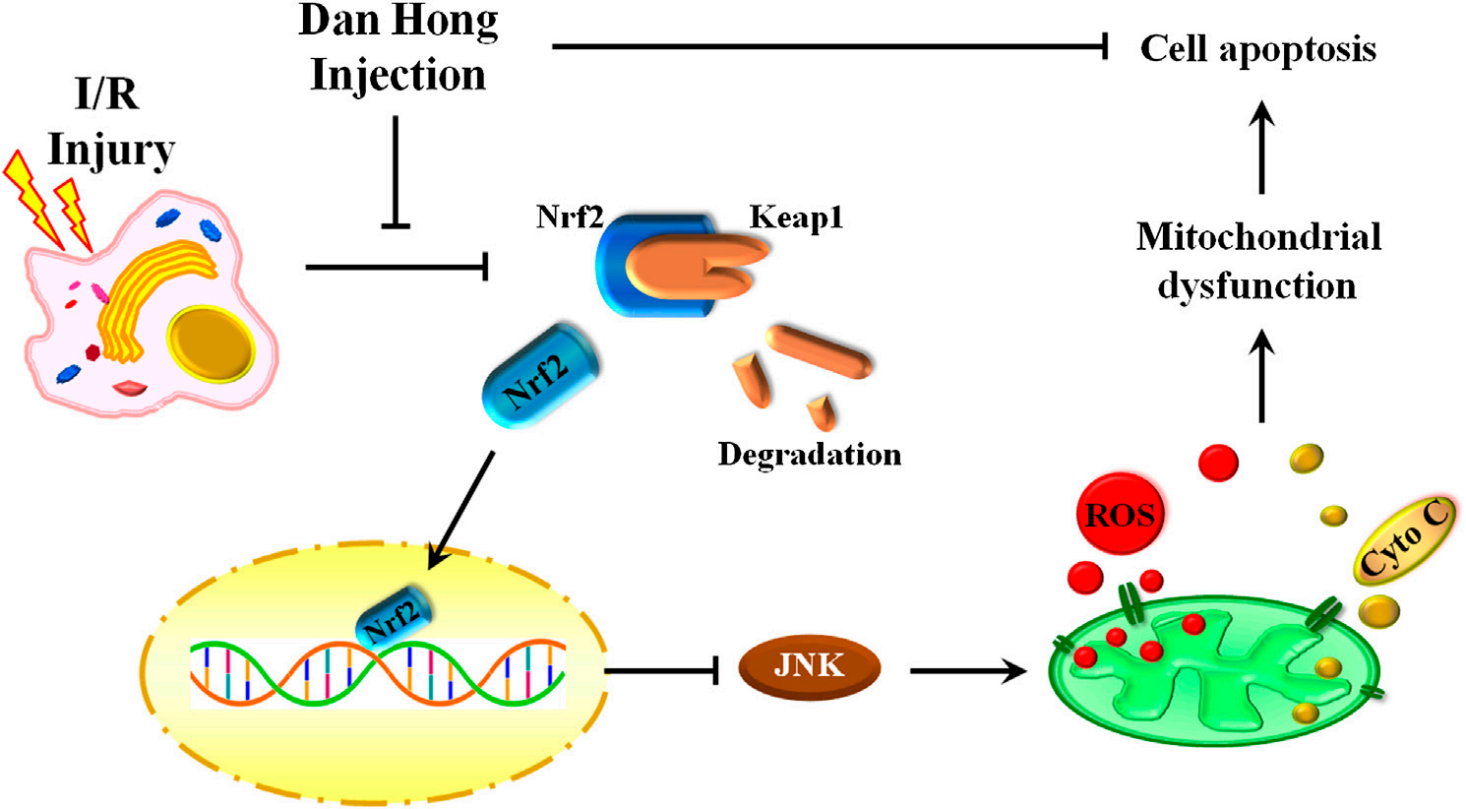
A schematic illustration showed that danhong injection protected mitochondrial integrity through the Keap1/Nuclear Factor Erythroid 2-Related Factor 2/JNK pathway in response to Hypoxia/Reoxygenation injury.

Our findings reveal an indispensable role of DHI in I/R treatment by regulation of mitochondrial integrity and an anti-apoptosis action. DHI degradedKeap1 and released Nrf2 to translocate to the nucleus. Then, DHI decreased JNK phosphorylation to maintain mitochondrial integrity and, finally, inhibited cytochrome c/caspase3-induced apoptosis (Figure 10). Thus, DHI may be an effective drug for maintaining mitochondrial integrity and treating myocardial I/R.

## Data Availability Statement

The raw data supporting the conclusions of this article will be made available by the authors, without undue reservation, to any qualified researcher.

## Ethics-Statement

The animal study was reviewed and approved by Zhejiang Chinese Medical University.

## Author Contributions

HW, YH and ZL conceived and designed the experiments and wrote the paper. ZL and YW performed research and contributed equally to this work. CL, CS, HZ. and JY. carried out the data analyses and interpreted the results.

## Funding

This work was supported by the grants from National Natural Science Foundation of China (No. 81874366 for HW; No. 81873226 for YH; No. 81803992 for YW), National Key R&D Program of China (No. 2017YFC1700400 for HW; No. 2017YFC1700403 for HW), Zhejiang Provincial Natural Science Foundation of Zhejiang Province (No. LZ17H270001 for JY), Key Laboratory of TCM Encephalopathy of Zhejiang Province (No. 2020E10012).

## Conflict of Interest

The authors declare that the research was conducted in the absence of any commercial or financial relationships that could be construed as a potential conflict of interest.
